# Biomimetic carbon monoxide delivery based on hemoglobin vesicles ameliorates acute pancreatitis in mice via the regulation of macrophage and neutrophil activity 

**DOI:** 10.1080/10717544.2018.1477860

**Published:** 2018-05-30

**Authors:** Kazuaki Taguchi, Saori Nagao, Hitoshi Maeda, Hiroki Yanagisawa, Hiromi Sakai, Keishi Yamasaki, Tomohiko Wakayama, Hiroshi Watanabe, Masaki Otagiri, Toru Maruyama

**Affiliations:** a Faculty of Pharmaceutical Sciences, Sojo University, Kumamoto, Japan;; b Department of Biopharmaceutics, Graduate School of Pharmaceutical Sciences, Kumamoto University, Kumamoto, Japan;; c Department of Chemistry, Nara Medical University, Kashihara, Japan;; d DDS Research Institute, Sojo University, Kumamoto, Japan;; e Department of Histology, Graduate School of Medical Sciences, Kumamoto University, Kumamoto, Japan;; f Center for Clinical Pharmaceutical Sciences, School of Pharmacy, Kumamoto University, Kumamoto, Japan

**Keywords:** Macrophage, polarization, carbon monoxide, acute pancreatitis, liposome

## Abstract

Macrophages play a central role in various inflammatory disorders and are broadly divided into two subpopulations, M1 and M2 macrophage. In the healing process in acute inflammatory disorders, shifting the production of M1 macrophages to M2 macrophages is desirable, because M1 macrophages secrete pro-inflammatory cytokines, whilst the M2 variety secrete anti-inflammatory cytokines. Previous findings indicate that when macrophages are treated with carbon monoxide (CO), the secretion of anti-inflammatory cytokine is increased and the expression of pro-inflammatory cytokines is inhibited, indicating that CO may have a potential to modulate the production of macrophages toward the M2-like phenotype. In this study, we examined the issue of whether CO targeting macrophages using a nanotechnology-based CO donor, namely CO-bound hemoglobin vesicles (CO-HbV), modulates their polarization and show therapeutic effects against inflammatory disorders. The results showed that the CO-HbV treatment polarized a macrophage cell line toward an M2-like phenotype. Furthermore, in an *in vivo* study using acute pancreatitis model mice as a model of an inflammatory disease, a CO-HbV treatment also tended to polarize macrophages toward an M2-like phenotype and inhibited neutrophil infiltration in the pancreas, resulting in a significant inflammation. In addition to the suppression of acute pancreatitis, CO-HbV diminished a subsequent pancreatitis-associated acute lung injury. This could be due to the inhibition of the systemic inflammation, neutrophil infiltration in the lungs and the production of HMGB-1. These findings suggest that CO-HbV exerts superior anti-inflammatory effects against inflammatory disorders via the regulation of macrophage and neutrophil activity.

## Introduction

The production of macrophages is related to the onset and progression of inflammatory disorders, because they secrete a variety of pro-inflammatory and anti-inflammatory mediators (Wynn et al., 2013). In general, macrophages can broadly undergo either classically M1 activation or alternative M2 activation (Sica & Mantovani, 2012). M1 and M2 macrophages are classified as such, by their cytokine production profiles: The former can produce many types of pro-inflammatory cytokines, such as tumor necrosis factor (TNF)-α, interleukin-1β (IL-1β) and interleukin-6 (IL-6), while the latter can produce anti-inflammatory cytokines, such as IL-10 (Sica & Mantovani, 2012). Since a better balance between M1 and M2 macrophages are desirable for the healing of inflammatory disorders, controlling macrophage polarization is the current focus of a potential target for designing a therapeutic strategy for the treatment of inflammatory disorders.

Carbon monoxide (CO) has attracted interest as a novel medical gas for use in the treatment of intractable disorders (Motterlini & Otterbein, 2010). This is because CO possesses potent cytoprotective effects because of its versatile biological functions such as anti-inflammatory, anti-oxidative and anti-apoptosis effects (Rochette et al., 2013). Otterbein et al. (2000) previously reported that a CO treatment inhibited the expression of pro-inflammatory cytokines, such as TNF-α and IL-1β, and increased the production of the anti-inflammatory cytokine, IL-10, in RAW 264.7 cells (a murine macrophage cell line) and in mice. Interestingly, after a CO treatment, cytokine production skewed the cytokine profiles secreted from M2 macrophages. These facts led us to the hypothesis that targeting of CO to macrophages might exert anti-inflammatory effects by skewing macrophage polarization in the direction/indirection of the M2 phenotype. However, in the absence of a carrier, controlling the disposition of CO molecules in the body is difficult, if not impossible.

Hemoglobin (Hb) is an ideal material for a carrier of CO by virtue of its ability to bind with CO molecules. However, using free Hb alone as a carrier is difficult because endogenous- or exogenous-derived Hb is not retained in blood for extended period of time and causes a number of adverse effects such as renal injury and hypertension (Savitsky et al., 1978; Buehler et al., 2010). As an innate biological system in the body, Hb is located inside red blood cells (RBC), which minimizes the risk of developing adverse effects and to prevent its rapid clearance from the body. Because of their convenience, RBC preparations are currently being used in clinics for the treatment of the patients with massive hemorrhages. Hence, RBC would be expected to be a practical Hb-based CO carrier. In fact, ∼75% of the CO stores in the body are found in the blood in the form of being to Hb in RBC. In cases of the development of hypoxia in tissues, CO stored in blood pool are supplied to these tissues (Coburn, 1970). It therefore appears that Hb in RBC acts as an endogenous CO storage and delivery cargo. However, some clinical problems for using RBC preparations remain, such as the potential of medical accidents (blood-type mismatching and uncertain infectious diseases etc) and short storage period (21–49 days in the world, depending on the country) (Flegel et al., 2014).

Based on the concept of a biomimetic delivery system (Parodi et al., 2017; Sheikhpour et al., 2017), Hemoglobin vesicles (HbV) represent a novel Hb-based CO carrier based on RBC-mimetic nanoparticles, in which a concentrated human Hb solution is encapsulated within a phospholipid vesicle (a liposome) with physicochemical properties similar to those of RBC, except for diameter (HbV; ca. 280 nm, and RBC; ca. 8000 nm) (Sakai, 2017). These RBC-mimetic structures of HbV have a good biocompatibility and relatively long half-life with Hb, even after a massive infusion (Sakai, 2017; Taguchi et al., 2017a). Furthermore, the phospholipid bilayer membrane that consists of a lipid membrane of HbV allows CO to penetrate, and then easily and stably bind to Hb in the form of HbV as well as RBC (Sakai et al., 2008a, b). Taking advantage of these favorable characteristics of HbV as a CO carrier, we previously developed CO-bound hemoglobin vesicles (CO-HbV) as a new type of CO donor (Taguchi et al., 2017b). In addition, we also found that a CO-HbV treatment suppressed the onset and progression of intractable inflammatory disorders in a mouse model via anti-inflammatory effects caused by either a decrease in the production of pro-inflammatory cytokines or an increase in the production of anti-inflammatory cytokine (Nagao et al., 2014, 2016a, 2016b). Thus, the controlled delivery of CO by CO-HbV might polarize macrophages toward an M2 phenotype in cases of inflammatory disorders, resulting in healing via the anti-inflammatory effects.

The aim of this study was to verify the contribution of anti-inflammatory effects via macrophage polarization after CO-HbV administration to therapeutic effects against inflammatory disorders. For this purpose, we first evaluate the release of CO from CO-HbV both *in vitro* and *in vivo*. We then examined the issue of whether CO-HbV was able to regulate M1/M2 polarization in vitro using RAW 264.7 cells. Finally, we evaluated whether CO-HbV could protect against the pathogenesis of inflammatory disorders via anti-inflammatory effects using a cerulein-induced acute pancreatitis model.

## Materials and methods

### Chemicals

Cerulein, lipopolysaccharide (LPS), and the anti-actin monoclonal antibody were purchased from Sigma-Aldrich (St Louis, MO). The antibiotics (10,000 U/ml penicillin, 10,000 μg/ml streptomycin) were purchased from nacalai tesque (Kyoto, Japan). Outdated donated human RBCs were provided by the Japanese Red Cross Society (Tokyo, Japan), and human Hb was purified through pasteurization and nanofiltration. The 1,2-dipalmitoyl-sn-glycero-3-phosphatidylcholine, cholesterol, and 1,5-O-dihexadecyl-*N*-succinyl-L-glutamate were purchased from Nippon Fine Chemical Co. Ltd. (Osaka, Japan). 1,2-distearoyl-sn-glycero-3-phosphatidyl-ethanolamine-N-PEG5000 was purchased from NOF Corp. (Tokyo, Japan). All other chemicals were of the highest grade commercially available, and all solutions were prepared using deionized water.

### Animals

All mice were purchased from Japan SLC, Inc. (Shizuoka, Japan). The mice were housed in a room with the temperature maintained at 18–24 °C and 40–70% relative humidity, with a 12-h light/dark cycle, and allowed free access to food and drinking water. Maintenance of the animals and the experimental procedures performed on them were carried out in accordance with NIH guidelines. All animal experiments were reviewed and approved by the Animal Care and Use committee of Kumamoto University (Permit #: A 27-003) and Sojo University (Permit #: 2014-P-018).

### Preparation of HbV and CO-HbV

HbV and CO-HbV were prepared according to previously reported procedures (Nagao et al., 2014). In short, the Hb solution was purified from outdated donated RBC, which was provided by the Japanese Red Cross Society (Tokyo, Japan), and the oxyhemoglobin converted into HbCO by bubbling with CO gas. The lipid bilayer of both the HbV and CO-HbV consisted of 1,2-dipalmitoyl-sn-glycero-3-phosphatidylcholine, cholesterol, and 1,5-O-dihexadecyl-N-succinyl-L-glutamate in a molar ratio of 5/4/0.9, and 1,2-distearoyl-sn-glycero-3-phosphatidyl-ethanolamine-N-PEG5000 (0.3 mol%). The CO-HbV particles were prepared by the extrusion method, and suspended in a physiological salt solution, filter-sterilized (Dismic, Toyo-Roshi, Tokyo, Japan; pore size, 450 nm). By illumination with visible light under an oxygen atmosphere, CO-HbV was converted to HbV. The HbV particles suspended in physiological salt solution bubbled with nitrogen for storage. The average diameters of the HbV and CO-HbV particles used in this study were maintained at ∼280 nm. The HbV and CO-HbV suspended in a physiological salt solution were at [Hb] = 10 g/dL and [lipid] = 9.0 g/dL. The carbonylHb content of the HbV and CO-HbV was less than 5% and nearly 100%, respectively.

### Assessment of CO releasing from CO-HbV *in vitro* and *in vivo*

Either HbV or CO-HbV was added to a 100% fetal bovine serum (FBS) in a glass vial ([Hb] = 1 g/dL), and immediately sealed and mixed at room temperature. At stipulated times, 1 mL of air in the head space of glass vial was collected, and the CO concentration was immediately determined using TRIlyzer mBA-3000 (Taiyo Instruments Inc, Osaka, Japan).

For *in vivo* analysis, male Sea-ICR mice (6 weeks old) were intravenously injected with CO-HbV (1000 mg Hb/kg), and venous blood samples were obtained at stipulated time after administration (0.08, 0.17, 0.5, 1, 2, 4, 8, and 12 h). The blood samples (0.1 mL) were mixed with 0.4 mL phosphate buffer saline and 10 μL saponin (16.7 mg/mL in 1 N H_2_SO_4_) to completely dissociate CO from Hb in a glass vial, and the glass vials were then sealed. After a 2-h incubation, 1 mL of air in the head space of glass vial was collected, and the CO concentration was immediately determined as described above.

### Cell experiment

RAW 264.7 cells were cultured in Dulbecco's modified eagle medium (DMEM) supplemented with 10% FBS, 100 μg/mL streptomycin, and 100 μg/mL penicillin at 37 °C, 5% CO_2_. The RAW 264.7 cells were seeded into six-well cell culture plates (500,000 cells/well). After incubation overnight, the cells were incubated with either M1 stimulant mixture [interferon (IFN)-γ (20 ng/mL), LPS (100 ng/mL)] or M2 stimulant mixture [interleukin-13 (IL-13, 20 ng/mL), interleukin-4 (IL-4, 20 ng/mL)] or HbV (5 mg Hb/mL) or CO-HbV (5 mg Hb/mL) for 24 h.

### Production of acute pancreatitis model mice and sample administration

Acute pancreatitis model mice were prepared as previously reported (Liu et al., 2016). The male C57BL/6N mice (6 weeks old) received a total of seven hourly injections of cerulein (i.p. 50 μg/kg), and LPS (10 mg/kg) was added to the last cerulein injection. Saline (10 mL/kg), HbV or CO-HbV was intravenously administered at a dose of 1000 mg Hb/kg at 30 min after the first cerulein injection.

### Quantitation of amylase, lipase activity and high mobility group box-1 (HMGB1) in plasma

Amylase and lipase activity were analyzed using automatic analyzer (Fuji DRI-CHEM 7000Z, Fujifilm, Tokyo, Japan). The amount of HMGB1 in the plasma was determined using an ELISA kit (Shino-Test, Tokyo, Japan).

### Hematoxylin and eosin (HE) stain and immunological staining

The collected pancreas and right lung were embedded in paraffin after fixing in 10% phosphate-buffered formalin. The slides (4-μm-thick sections) were stained with HE, or treated with the primary antibodies containing myeloperoxidase (MPO) or nitrotyrosine (NO_2_-Tyr) as previously described (Nagao et al., 2016b).

### Quantification of cytokines and HMGB1 in the pancreas

The collected pancreas samples were homogenized using a homogenizer in PBS buffer contained EDTA (10 mM), protease inhibitor cocktail (1%), and Tween-20 (0.05%). The supernatant was used for analysis after centrifuging (21,000 g, 10 min, 4 °C) the samples twice. The amounts of TNF-α, IL-6, IL-1β, and IL-10 in the pancreas were determined using an ELISA kit (Biolegend, San Diego). The amount of HMGB1 in pancreas was determined using an ELISA kit (Shino-Test, Tokyo, Japan).

### Evaluation of the lung wet/dry ratio

The lung wet/dry ratio was determined using one lobe of the left lung as reported previously (Nagao et al., 2016b).

### Real time RT-PCR analysis

The levels of mRNA expression were evaluated by real time RT-PCR analysis as previously reported (Ogaki et al., 2015). The following PCR primers were used in present study. GAPDH forward 5′-AACTTTGGCATTGTGGAAGG-3′, reverse 5′-ACACATTGGGGGTAGGAACA-3′; TNF-α forward 5′-CATGAGCACAGAAAGCATGATCCG-3′, reverse 5′-AAGCAGGAATGAGAAGAGGCTGAG-3′; NOS2(iNOS) (for *in vitro*) forward 5′-AGTCAACTGCAAGAGAACGGA-3′, reverse 5′-GAAGAGAAACTTCCAGGGGCA-3′; NOS2 (iNOS) (for *in vivo*) forward 5′-GTGGTGACAAGCACATTTGG-3′, reverse 5′-AAGGCCAAACACAGCATACC-3′; CD163 forward 5′-ATGGGCTAACTCCAGCGCCG-3′, reverse 5′-GATCCATCTGAGCAGGTCACTCCA-3′; MRC1 (CD206) forward 5′-GCCAGAGACATAACAGCA-3′, reverse 5′-CAGGTTTCCTTTCAGTCCT-3′; IL-10 forward 5′-CTGGACAACATACTGCTAACCG-3′, reverse 5′-GGGCATCACTTCTACCAGGTAA-3′.

### Statistics

Significant differences among groups were performed by variance analysis (ANOVA) followed by the Bonferroni analysis. A probability value of *p* < .05 was considered to indicate statistical significance.

## Results

### CO releasing from CO-HbV *in vitro* and *in vivo*

**Figure 1.**
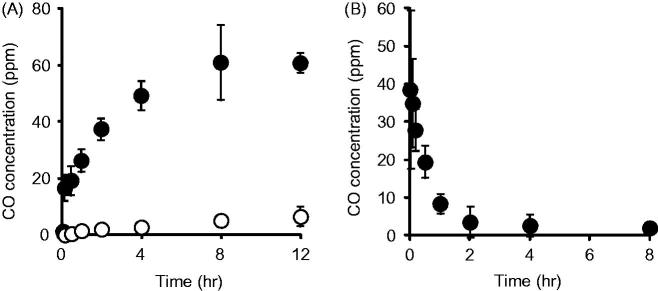
(A) CO release from HbV (open circle) or CO-HbV (closed circle) in the presence of 100% FBS. Either HbV or CO-HbV was mixed with 100% FBS in a glass vial. At stipulated time after mixing, the CO concentration in the head space of the glass vial was determined using gas chromatography with a CO-analyzer. Data are mean ± SD (*n* = 3 per group). (B) CO concentration in blood after CO-HbV administrating to healthy mice. Mice were single injected CO-HbV, and CO concentrations in blood were measured using CO analyzer. Data are mean ± S.D. (*n* = 4).

The CO release from CO-HbV was first evaluated *in vitro* using gas chromatography. As shown in Figure 1(A), CO-bund HbV continuously released CO for a period of 8 h in FBS, while the release of CO from HbV was negligible. In addition, the CO concentration in the blood after CO-HbV administrating to healthy mice was also assessed. The results showed that the CO concentration derived from CO-HbV gradually decreased in the blood and reached nearly zero at 8 h after administration (Figure 1(B)). Thus, CO-HbV would be expected to act as a long-lasting CO donor without CO being accumulated in the body.

### Effect of CO-HbV on macrophage polarization in RAW 264.7 cells

**Figure 2.**
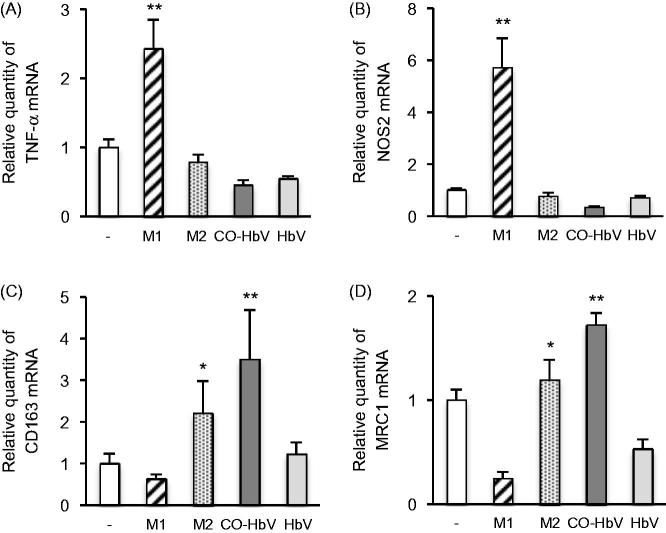
The changes of mRNA expression of (A) TNF-α, (B) NOS2, (C) CD163 and (D) MRC1 after incubation with either M1-trophic cytokines (LPS and IFN-γ), M2-trophic cytokines (IL-13 and IL-4), CO-HbV or HbV in RAW 264.7 cells. TNF-α and NOS2: M1 macrophage marker, CD163 and MRC1: M2 macrophage marker. Error bars indicate the S.E. of six separate experiments. *p < .05,**p < .01 versus non-treatment.

Either HbV, CO-HbV or classically defined stimuli (M1-trophic cytokines; LPS and IFN-γ, M2-trophic cytokines; IL-13 and IL-4) was added to RAW 264.7 cells, and then the expression of major M1 (TNF-α and NOS2) and M2 markers (CD163 and MRC1) were evaluated. As a result, the mRNA levels of M2 macrophage markers were significantly increased by the CO-HbV treatment as well as the M2-trophic cytokines treatment (Figure 2(C) and (D)). On the other hand, the mRNA levels of M1 macrophage markers were found to be significantly increased only by the M1-trophic cytokines treatment but the CO-HbV treatment had no effect (Figure 2(A) and (B)). In addition, the levels of the M1 nor M2 macrophage marker remain unchanged, when RAW 264.7 cells were incubated with HbV. Thus, CO derived from CO-HbV has the potential to polarize macrophages toward M2-like phenotypes.

### Biological and histological responses after CO-HbV treatment in acute pancreatitis model mice

**Figure 3.**
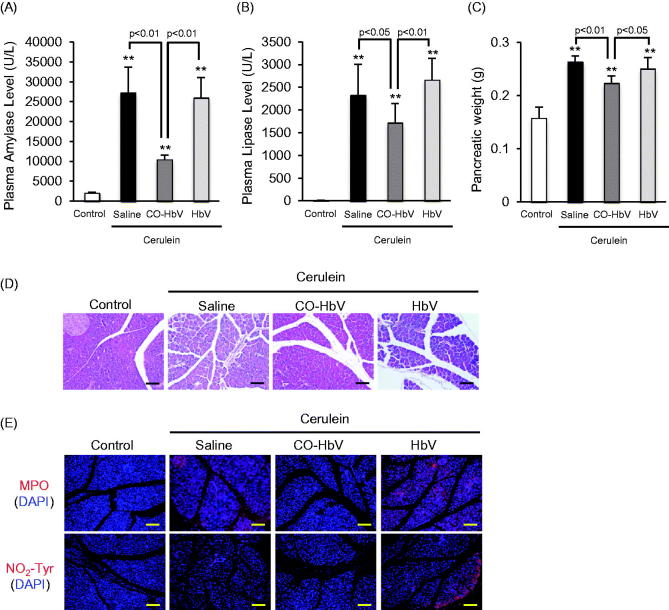
The plasma levels of (A) amylase, (B) lipase and (C) pancreatic weight after CO-HbV administrating to acute pancreatitis model mice. Data are mean ± S.D. (*n* = 6 per group). **p < .01 versus control. (D) Micrographs of pancreas slides stained with HE. (E) Immunological staining of pancreatic slices for MPO (red, upper) and NO_2_-Tyr (red, lower). Blue staining represents the nuclei immunostained with DAPI (counterstain). Scale bars: 100 μm.

Macrophages are associated with the pathogenesis of acute pancreatitis because they are a population of inflammatory cells (Shrivastava & Bhatia, 2010). Therefore, acute pancreatitis model mice induced by cerulein were used in an *in vivo* study of the contribution of CO-HbV to anti-inflammatory effects including macrophage polarization. Initially, therapeutic effects of saline, HbV or CO-HbV on acute pancreatitis were assessed in acute pancreatitis model mice. The results clearly showed that the CO-HbV treatment significantly suppressed an elevation of pancreatic enzyme levels in the plasma (Figure 3(A) and (B)). In addition, CO-HbV treatment also showed the lower pancreatic weight compared to saline and HbV treatment (Figure 3(C)). Furthermore, the pancreatic protective effects were observed by light micrographs only in mice treated with CO-HbV (Figure 3(D)). Since neutrophils-derived reactive oxygen species (ROS) are attributed to induce pancreatitis, the extent of neutrophil infiltration was evaluated by the means of the immunological staining of the pancreatic sections for MPO, which is general marker of neutrophil (Leung & Chan, 2009). As shown in Figure 3(E), CO-HbV treatment observed less MPO accumulation in pancreas of acute pancreatitis model mice than saline and HbV treatment. Accompanying the MPO accumulation, a substantial accumulation of NO_2_-Tyr, a maker of oxidative injury, were observed in saline and HbV treatment group but not in CO-HbV group (Figure 3(E)).

### Macrophage polarization in pancreas of acute pancreatitis model mice

**Figure 4.**
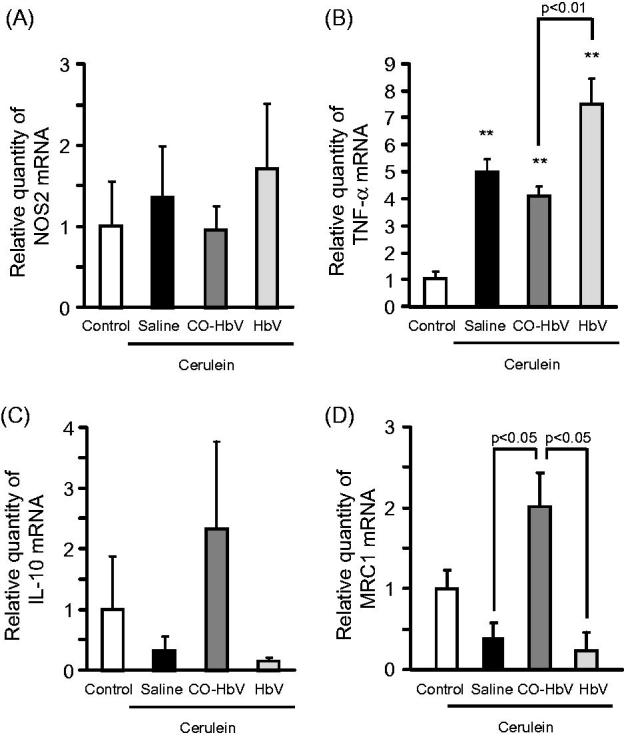
M1 and M2 polarization of macrophages in the pancreas of acute pancreatitis model mice. The mRNA expressions of (A) NOS2, (B) TNF-α, (C) IL-10, and (D) MRC1 were determined using pancreas tissue collected at 12 h after the start of cerulein administration. Data are mean ± S.E. (*n* = 6 per group). **p < .01 versus control.

The mRNA level of M1 macrophage markers and M2 macrophage markers in the pancreas of acute pancreatitis model mice were evaluated. As a result, the mRNA expressions of NOS2 and TNF-α (M1 macrophage markers) were relatively higher levels in saline- and HbV-treated pancreatitis mice than CO-HbV-treated pancreatitis mice (Figure 4(A) and (B)), whilst the mRNA expression of both IL-10 and MRC1 (M2 macrophage markers) showed higher levels in mice treated with CO-HbV than saline- or HbV-treated mice (Figure 4(C) and (D)). These results conclude that CO-HbV prevents the development of acute pancreatitis via skewing macrophage production toward an M2-like phenotype.

### Cytokine levels in the pancreas of acute pancreatitis model mice

**Figure 5.**
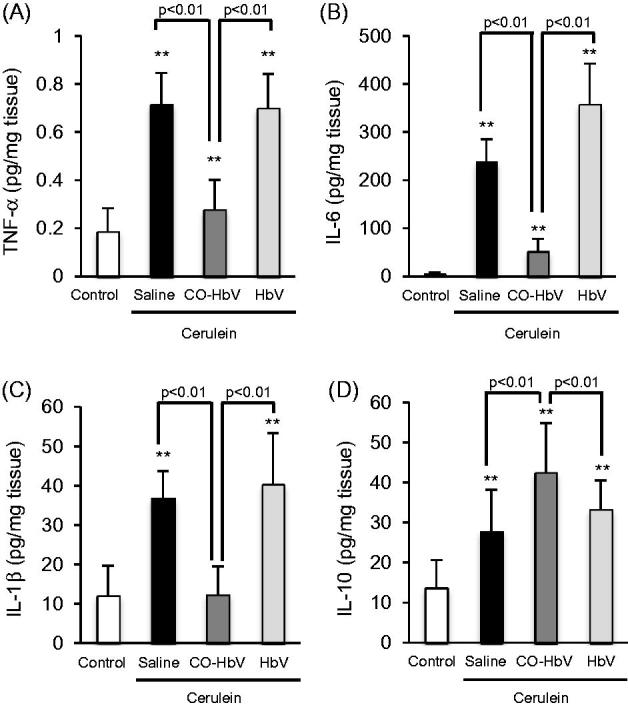
The levels of (A) TNF-α, (Β) IL-6, (C) IL-1β, and (D) IL-10 in the pancreas of acute pancreatitis model mice. Pancreas tissue collected at 12 h after the start of cerulein administration. Data are mean ± S.D. (*n* = 6 per group). **p < .01 versus control.

M1 macrophages are able to secrete pro-inflammatory mediators, whilst M2 macrophages secrete anti-inflammatory mediators (Sica & Mantovani, 2012). Thus, we evaluated the representative pro- and anti-inflammatory cytokines production in the pancreas of acute pancreatitis model mice. As shown in Figure 5(A)–(C), the saline and HbV treatment showed a dramatic increased in the production of TNF-α, IL-6, and IL-1β compared to the CO-HbV treatment. On the other hand, CO-HbV treatment suppressed the pro-inflammatory cytokines production (Figure 5(A)–(C)) and promoted the anti-inflammatory cytokines production in pancreatitis model mice (Figure 5(D)).

### Evaluation of pancreatitis-associated distant lung injury after CO-HbV treatment in acute pancreatitis model mice

**Figure 6.**
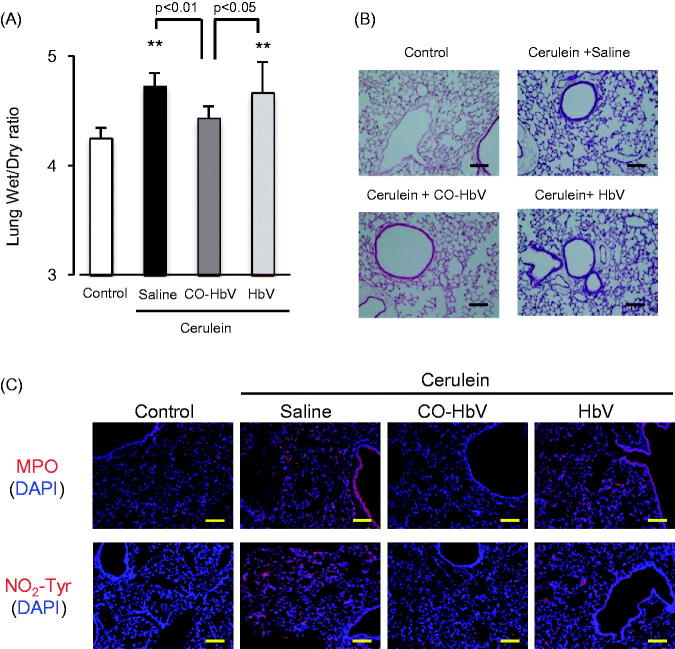
Evaluation of distant lung injury after CO-HbV treatment in acute pancreatitis model mice. (A) Lung wet/dry ratio were determined using one lobe of the left lung collected at 12 h after the start of cerulein administration. Data are mean ± S.D. (*n* = 6 per group). **p < .01 versus control. (B) Micrographs of lung section stained with HE and (C) immunological staining of lung slices for MPO (red, upper) and NO_2_-Tyr (red, lower). Blue staining represents the nuclei immunostained with DAPI (counterstain). Scale bars: 100 μm.

Inflammatory cell associated with pancreatitis promote, not only intrapancreatic injury, but also extrapancreatic multiorgan injury. In fact, acute pancreatitis frequently induces acute lung injury as a major complication (Zhou et al., 2010). Thus, the effects of CO-HbV on lung injury, one type of distal organ injury, were also investigated in the acute pancreatitis mice. The findings indicate that the CO-bond HbV treatment decreased the lung wet/dry ratio, which reflects edema, in acute pancreatitis mice (Figure 6(A)) and morphological changes (Figure 6(B)), suggested that CO-HbV attenuate the extent of lung injury. In addition, the results of immunological staining showed that CO-HbV treatment also decreased the MPO accumulation (neutrophil infiltration) and NO_2_-Tyr accumulation (oxidative product) of acute pancreatitis mice (Figure 6(C)), indicating that CO-HbV attenuated the inflammation and oxidative injury in lungs.

### Effect of CO-HbV on HMGB1 levels in pancreatic and plasma of acute pancreatitis model mice

**Figure 7.**
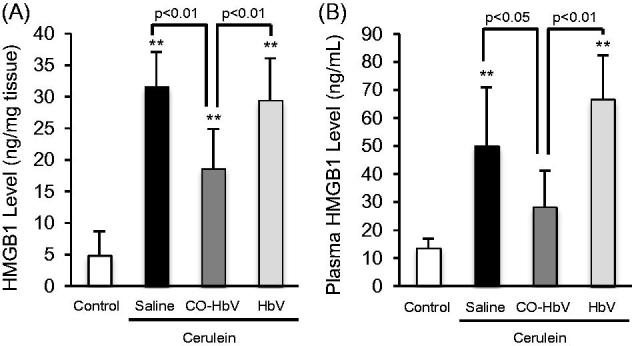
Pancreatic and plasma HMGB1 levels in acute pancreatitis model mice. HMGB1 level in (A) the pancreas and (B) plasma were determined using pancreas and plasma collected at 12 h after the start of cerulein administration. Data are mean ± S.D. (*n* = 6 per group). **p < .01 versus control.

Since damage-associated molecular patterns, such as HMGB1, contribute to linking between acute pancreatitis and distal organ injuries (Gukovskaya et al., 2017), we also evaluated HMGB1 levels in both the pancreas and plasma of the acute pancreatitis model mice. The HMGB1 level in pancreas and plasma were significantly increased by the induction of acute pancreatitis. However, the CO-HbV treatment significantly suppressed the levels of HMGB1 in both the pancreas and plasma compared to the saline treatment, but HbV treatment had no effect (Figure 7).

## Discussion

Controlling the balance between the production of pro-inflammatory and anti-inflammatory cytokines is a key factor in the healing process in a number of disorders. In the case of inflammatory disorders, it appears that anti-inflammatory cytokines, such as IL-10, function in a positive manner regarding healing. Based on our knowledge, M1/M2 macrophages are widely associated with the regulation of inflammation via the different cytokine expression profiles: M1 macrophages represent a source of pro-inflammatory cytokines, while M2 macrophage represent a source of anti-inflammatory cytokines (Sica & Mantovani, 2012). Accordingly, the skewing of macrophage polarization toward either the production of M1 or M2 macrophages would likely significantly advance therapeutic strategies that involve the treatment of inflammatory disorders in pharmaceutics and medicine (Alvarez et al., 2016). In this study, CO-HbV would have the ability to skew macrophage polarization toward an M2-like phenotype and not an M1 phenotype in RAW 264.7 cells (Figure 2). In addition, the CO contained in CO-HbV could be released from HbV, both *in vitro* and *in vivo* (Figure 1). These interesting results suggest that CO-HbV could be used to modulate macrophage production to an M2-like phenotype even under pathological conditions, thus promoting the healing of M2 macrophage-related pathology such as inflammatory disorders via the anti-inflammatory effects.

Macrophages are associated with an altered cytokine reaction in acute pancreatitis which may constitute the basis of onset and progression (Shrivastava & Bhatia, 2010). Yu et al. (2016) recently reported on the contribution of M2 macrophages in repairing pancreatic tissue in the early period of an acute pancreatitis model rat. Therefore, it is possible that CO-HbV would shows therapeutic effects against acute pancreatitis due to the regulation of macrophage polarization toward M2-like macrophages. As expected, the level of a marker of M2 macrophages in the pancreas tended to increase in pancreatitis mice that were treated with CO-HbV, but the marker for M1 macrophages did not (Figure 4). Accompanying the change of macrophage behavior to an M2-like phenotype, CO-HbV suppressed the production of pro-inflammatory cytokines in the pancreas, while HbV showed the same cytokine production profiles as the saline treatment (Figure 6). This modulation of macrophage behavior as a result of the CO-HbV treatment could, in part, contribute to a significant reduction in the intensity of pancreatic injury (Figure 3).

HMGB1 is an endogenous ligand of the Toll-like receptor (TLR)-4, the signals of which generate the production of pro-inflammatory cytokines and mediate necrosis-induced sterile inflammation (Erridge, 2010; Andersson & Tracey, 2011). Su et al. (2016) using autoimmune myocarditis model mice, recently demonstrated that HMGB1 facilitated macrophage polarization toward an M1-like phenotype and that this process was dependent on the TLR-4-PI3Kγ-Erk1/2 pathway. Therefore, HMGB1 would be expected to play a crucial role in the inflammatory response of inflammatory disorders via TLR-4 stimulation. In this study, CO-HbV caused a decrease in HMGB1 levels in both the pancreas and plasma (Figure 7). In addition, although we did not evaluate whether CO-HbV effected TLR-4-mediated responses, it was reported that CO donors inhibit the activation or expression of TLR-4 under pathological conditions (Xue & Habtezion, 2014; Ogaki et al., 2015). Therefore, CO-HbV may decrease the pathogenesis of acute pancreatitis through the HMGB1/TLR-4 pathway.

Neutrophil infiltration in the pancreas occurs in the early stage of acute pancreatitis, followed by macrophage recruitment (Montecucco et al., 2014). Although neutrophils appear to be related to the progression of acute pancreatitis via multiple pathways, they act as important generators of ROS and are responsible for local pancreatic damage (Leung & Chan, 2009). The findings reported herein indicate that the CO-HbV treatment attenuated the accumulation of neutrophils in pancreatic tissue in ceruleine-induced acute pancreatitis model mice (Figure 3(E)). Furthermore, corresponding to MPO accumulation, immunostaining for NO_2_-Tyr clearly showed that CO-HbV inhibited oxidative injury in the pancreas (Figure 3(E)). Based on these findings, it appears that the inhibition of the infiltration of neutrophils by a CO-HbV treatment would partly contribute to the suppression of the progression of acute pancreatitis.

During acute pancreatitis, the systemic inflammatory response syndrome (SIRS) typically causes an acute lung injury, and leads to the development of an acute respiratory distress syndrome, finally resulting in an increased mortality. Therefore, the suppression of both local inflammatory and subsequent lung injury would be expected to rescue patients with acute pancreatitis. In this study, CO-HbV ameliorated, not only acute pancreatic injury but also acute lung injury in a cerulein-induced acute pancreatitis model (Figure 6). Although the exact mechanisms responsible for pancreatitis-related acute lung injury are not fully understood, an accumulating body of evidence suggests that the following factors are related to the onset and progression of this complication in acute pancreatitis. One factor is the infiltration of neutrophils into tissues within the lungs. As well as in the pancreas, neutrophils appear to be recruited to the lung in the early stage of pancreatitis and cause ROS-related injuries in the lungs (Montecucco et al., 2014). Another factor is the production of pro-inflammatory cytokines (Bhatia & Moochhala, 2004). Pro-inflammatory cytokines, such as TNF-α, IL-6, and IL-1β, that are released during the course of pancreatitis mediate the pathogenesis of SIRS including lung injury. In fact, it was reported that IL-6 levels in the circulation are excellent predictors of the severity of lung injury in the etiology of acute pancreatitis (Leser et al., 1991). Additionally, HMGB1 is also key factor in the development of the acute lung injury. Luan et al. (2013) previously reported that the downregulation of HMGB1 protected against the development of acute lung injury associated with acute pancreatitis via inhibiting NF-κB activation in the lungs. Furthermore, HMGB1 can stimulate the release of pro-inflammatory cytokines, such as TNF-α and IL-1β, and vice versa (Wang et al., 1999a, b). These facts imply that HMGB1 contributes to the pathogenesis of acute pancreatitis-associated SIRS, including acute lung injury. In this study, CO-HbV was found to effectively inhibit (i) the infiltration of neutrophils into lungs and subsequent oxidative injury (Figure 6(C)), (ii) the production of pro-inflammatory cytokines in the pancreas (Figure 5), and (iii) the levels of HMGB1 in both the pancreas and the circulation (Figure 7). These versatile effects of CO-HbV on systemic inflammation may be attributed to the protective effects against acute lung injury associated with acute pancreatitis.

It is well known that CO exerts cytoprotective effects against inflammatory disorders when the CO concentration in the body is low (∼500 ppm). On the other hand, a high concentration of CO acts as a toxic gas with deleterious symptoms, which become worse with increasing CO concentration and can ultimately lead to death (Roderique et al., 2015). Hence, the safety profile of CO-HbV needs to be taken into account. In the pharmacokinetics of CO after CO-HbV administration at a dose of 1000 mg Hb/kg, the maximum CO concentration in the blood was much lower than the toxic concentration of CO (>500 ppm) (Figure 1(B)), indicating that CO-HbV administration of doses up to 1000 mg Hb/kg could not lead to CO poisoning. In addition, our previous study using a hemorrhagic shock model rat showed that the CO derived from CO-HbV was completely exhaled within 6 h after administration unless the lungs failed to function (Sakai et al., 2009), indicating that CO would not accumulate in the body after CO-HbV administration. We also previously reported the CO-HbV showed no severe adverse effects including death, and clinical laboratory tests and histopathological changes remained normal for 28 days (Nagao et al., 2016a). Taken together, the safety profiles of HbV such as its biological compatibility, the absence of toxicity, the absence of accumulation in the body (Sakai, 2017; Taguchi et al., 2017a), CO-HbV possesses the favorable safety characteristics for use as a CO donor and as a contributor to the therapy against inflammatory disorders. However, it has been still remained some limitations in present study. One is that the detail disposition of CO after/before releasing from CO-HbV is unclear. Another is that it is not clarified whether CO-HbV effects on the immune cells in the blood stream prior to their arrival at the injury site. Since clarifying these issues would be helpful to understand the mechanism of anti-inflammatory effects of CO-HbV, further study will be needed in the future.

## Conclusions

M2 macrophages play a role in the healing process of various disease, indicating that the modulation of macrophages toward an M2 macrophage is of current interest in terms of developing a strategy in pharmaceutics and medicines. The findings of the present study show that CO-HbV can skew the macrophage polarization toward an M2-like phenotype. Therefore, CO-HbV has the potential for use as a therapy for M2 macrophage-related pathology such as atherosclerosis and diabetes. Furthermore, it should be noted that this is first report to demonstrate the effect of CO on macrophage polarization. Thus, these findings suggest a novel anti-inflammatory pathway for CO in inflammatory disorders.
